# Oceanic records of North American bats and implications for offshore wind energy development in the United States

**DOI:** 10.1002/ece3.8175

**Published:** 2021-10-11

**Authors:** Donald I. Solick, Christian M. Newman

**Affiliations:** ^1^ Vesper Bat Detection Services Fort Collins Colorado USA; ^2^ Electric Power Research Institute Palo Alto California USA

**Keywords:** Atlantic Ocean, bats, North America, offshore, Pacific Ocean, wind energy, wind turbines

## Abstract

Offshore wind energy is a growing industry in the United States, and renewable energy from offshore wind is estimated to double the country's total electricity generation. There is growing concern that land‐based wind development in North America is negatively impacting bat populations, primarily long‐distance migrating bats, but the impacts to bats from offshore wind energy are unknown. Bats are associated with the terrestrial environment, but have been observed over the ocean. In this review, we synthesize historic and contemporary accounts of bats observed and acoustically recorded in the North American marine environment to ascertain the spatial and temporal distribution of bats flying offshore. We incorporate studies of offshore bats in Europe and of bat behavior at land‐based wind energy studies to examine how offshore wind development could impact North American bat populations. We find that most offshore bat records are of long‐distance migrating bats and records occur during autumn migration, the period of highest fatality rates for long‐distance migrating bats at land‐based wind facilities in North America. We summarize evidence that bats may be attracted to offshore turbines, potentially increasing their exposure to risk of collision. However, higher wind speeds offshore can potentially reduce the amount of time that bats are exposed to risk. We identify knowledge gaps and hypothesize that a combination of operational minimization strategies may be the most effective approach for reducing impacts to bats and maximizing offshore energy production.

## Graphical abstract

Much has been learned about bat interactions with land‐based turbines in North America over the past 15 years, but little is known about potential risks and impacts to bats in the maritime environment despite a sociopolitical push to develop this resource. We synthesize historic and contemporary records of bats observed and acoustically recorded in North American waters to gain a better understanding of the offshore occurrence and behavior of North American bats and to place this in the context of offshore wind energy development in the United States. We aim to elucidate which North American bat species are most at risk, when and where we might expect the highest fatalities assuming the risk to bats by offshore is similar to terrestrial wind energy development, and whether aspects of offshore bat behavior can help inform mitigation strategies for reducing impacts by offshore wind energy development.

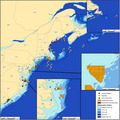

## 1 INTRODUCTION

**FIGURE 1:**
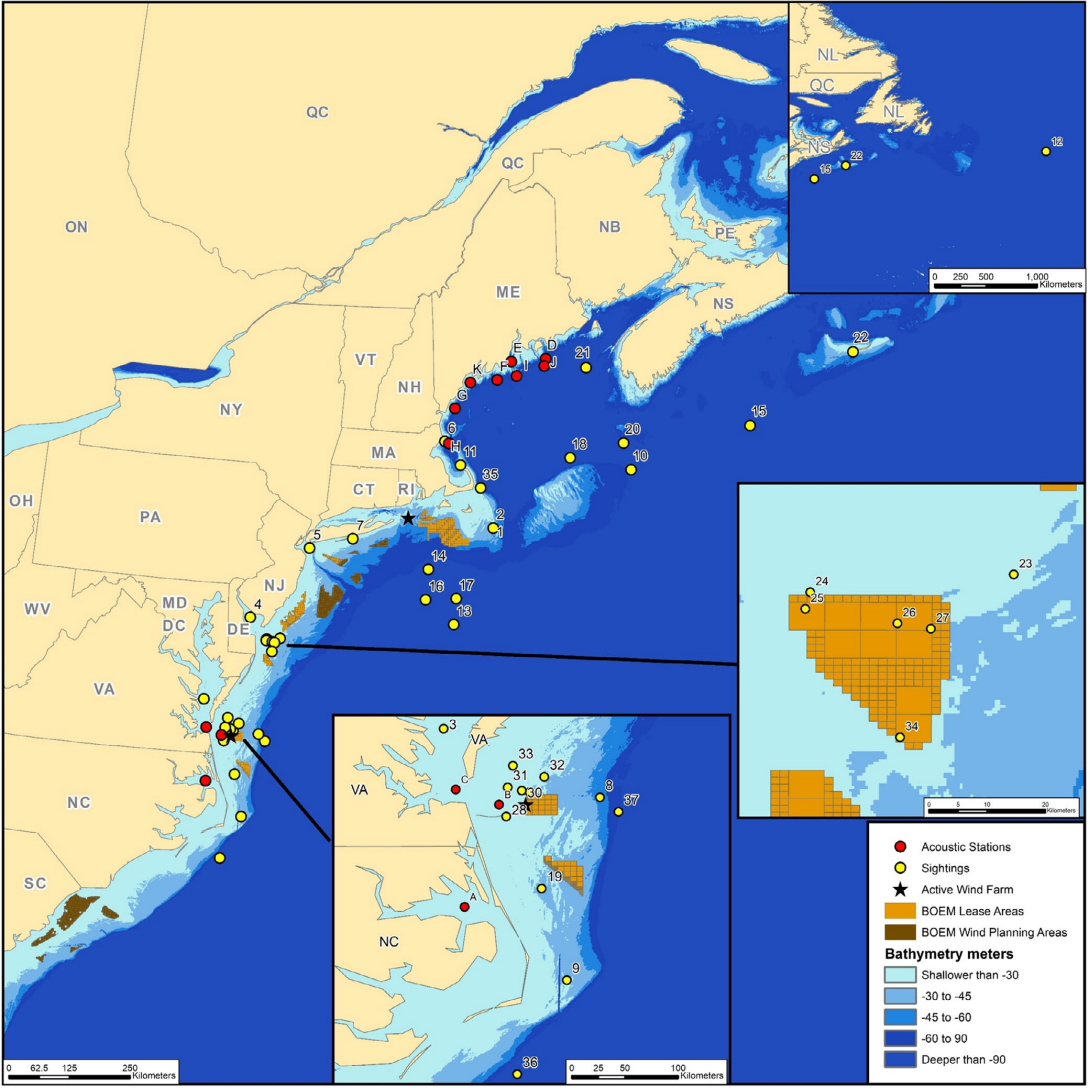
Distribution of bat sightings and acoustic recording locations in the Atlantic Ocean, in relation to operating wind energy facilities and leased areas. Numbered sightings and lettered recording locations are described in Tables S1 and S2. BOEM, Bureau of Ocean Energy Management

The electricity potential from offshore wind in the United States is estimated to be more than 2000 gigawatts, roughly twice the nation's current total generation (Musail et al., 2016). Two wind farms are now in operation off the coasts of Rhode Island and Virginia (Figure 1), while 29 offshore wind farms are in varying stages of development in the United States (AWEA, 2020a), with a projected build‐out of 30 gigawatts of offshore energy by the year 2030. The adverse effects of offshore wind generation on wildlife are generally acknowledged to be low relative to those of conventional electricity generation technologies (Allison et al., 2019; Gibson et al., 2017). However, adverse impacts are still possible, and understanding the ecological significance of these effects is necessary for responsible development of offshore wind energy resources.

There is growing concern that North American bat populations are being adversely impacted by land‐based wind development. An estimated 600,000 to 888,000 bats died from interactions with land‐based wind turbines in the United States during 2012 (Hayes, 2013; Smallwood, 2013), and installed wind power capacity has nearly doubled over the following eight years (AWEA, 2020b; Orrell et al., 2013). Some North American species, such as the hoary bat (*Lasiurus cinereus*), can potentially be at risk of population decline or extinction due to wind energy development (EPRI, 2020; Frick et al., 2017). Bat fatalities are mainly due to collisions with moving turbine blades (Grodsky et al., 2011; Lawson et al., 2020; Rollins et al., 2012), though the underlying reasons for why bats approach turbines are still largely unknown (Barclay et al., 2017; Cryan & Barclay, 2009). To date, postconstruction monitoring studies of land‐based wind energy facilities in the United States indicate the following: (a) long‐distance migrating species (e.g., hoary bat, eastern red bat [*Lasiurus borealis*], and silver‐haired bat [*Lasionycteris noctivagans*]) compose approximately 72% of reported bats killed; (b) the majority of fatalities occur during the autumn migration season (August and September); and (c) most fatalities occur on nights with relatively low wind speeds (e.g., <6.0 m/s; Arnett et al., 2008; AWWI, 2020).

Bats are primarily associated with terrestrial environments, yet some species are known to forage or migrate offshore. In Europe, field observations and recaptures of marked bats have shown that some species migrate seasonally across the Baltic and North Seas between the European continent and either Sweden or the United Kingdom, and some nonmigratory species forage over water far from shore (Ahlén et al., 2007, 2009; Hüppop & Hill, 2016; Lagerveld et al., 2014; Moores, 2017). In general, bats have been observed flying over large bodies of water (Hatch et al., 2013; Murphy & Nichols, 1913; Nichols, 1920), landing on ships at sea (Brown, 1953; Carter, 1950; Esbérard & Moreira, 2006; Griffin, 1940; Haagner, 1921; Mackiewicz & Backus, 1956; Norton, 1930; Peterson, 1970; Thomas, 1921; Van Deusen, 1961), roosting on gas and oil platforms (Boshamer & Bekker, 2008), arriving on remote islands (Allen, 1923; Cryan & Brown, 2007; Hitchcock, 1943; Paracuellos et al., 2020; Petersen et al., 2014; Tenaza, 1966; Van Gelder & Wingate, 1961), or otherwise encountered in areas or situations suggesting the animals traveled over large bodies of water (Maunder, 1988; Merriam, 1887; Miller, 1897; Saunders, 1930). Acoustic, radar, and high‐altitude videography surveys in the Gulf of Maine (Peterson et al., 2014, 2016) and in the Mid‐Atlantic (Craven et al., 2020; Geo‐Marine, 2010; Hatch et al., 2013; Peterson et al., 2016; Sjollema et al., 2014) have revealed some offshore bat activity patterns and behavior in North America. Despite these efforts, the frequency and extent of seasonal bat foraging and migration activities in the North American marine environment is poorly understood, and the degree to which bat populations can potentially be impacted by offshore wind development is largely unknown.

## 2 METHODS

We aim to elucidate which North American bat species have the highest exposure risk from offshore wind development, and when and where we might expect the highest fatalities to occur at sea. We also assess whether observations of offshore bat behavior can help inform minimization strategies for reducing impacts by offshore wind energy development. We synthesize information from historic oceanic records and more contemporary records to gain a better understanding of the offshore occurrence and behavior of North American bats and to place this in the context of offshore wind energy development in the United States. We exclude data on bat use of the nonoceanic Great Lakes (e.g., McGuire et al., 2012) from our review, although some of the patterns and behaviors described here may be applicable to bat use of inland lakes.

To best characterize bat migratory behavior over the open ocean, we summarize data on sightings of bats at sea and of acoustic recordings collected at offshore structures, such as buoys, towers, and lighthouses on barren, wave‐swept rocks. Coastal data (e.g., Moore, 2015; Moore & Best, 2018) and records from islands containing vegetation (e.g., Dowling, 2018; Dowling & O’Dell, 2018; Dowling et al., 2017; Johnson & Gates, 2008; Johnson et al., 2011; Peterson et al., 2014, 2016) were not included in our data summaries because these features likely harbor resident populations of bats and offshore wind development is not planned for these habitats. Use of the marine environment by bats has been studied more extensively in European waters, including surveys at operating offshore wind farms, and we incorporate information from these studies to provide framework for the behavior of North American species.

Several biases are inherent to this review. Sightings of bats flying over the ocean are necessarily restricted to daytime hours and to an observer's viewshed, so most sightings are reported during daylight hours and within a few dozen meters. As well, most sightings reported here were opportunistic, made while observers were engaged in other activities. Acoustic surveys can capture nighttime bat activity and are typically more rigorous and systematic than sightings from boats, but recordings are limited to the range of the detector—approximately 30 m for most species (Adams et al., 2012)—and require that bats actively echolocate while flying over the water. There is evidence that some European species echolocate over water (Ahlén et al., 2009), but hoary bats in North America are capable of making inconspicuous echolocation calls or flying without echolocating at all (Corcoran et al., 2021; Corcoran & Weller, 2018). Population impacts due to collision with offshore turbines are also impossible to assess because the population size of species likely to collide with offshore turbines is unknown, as is the proportion of those populations that flies over the ocean, or that might encounter turbine blades. Despite these limitations to the data, we think a thorough examination of North American oceanic records is needed as a starting point for understanding potential exposure risks to bats in the offshore environment and identifying knowledge gaps moving forward.

## 3 RESULTS AND DISCUSSION

### 3.1 Spatial distribution

All records of North American bats flying over the open ocean have occurred in the Atlantic region between North Carolina and Nova Scotia (Tables S1 and S2; Figure 1). When specified, bats were visually observed flying over open water or landing on ships at sea between 2.6 and 817.3 km from the nearest land (*n* = 37 records; median = 39.2 km; Table S1; Figure 1). Acoustic surveys in the Mid‐Atlantic and Gulf of Maine recorded bats at various offshore structures (e.g., buoys, lighthouses) between 5.9 and 41.6 km from land (Table S2; Figure 1; Peterson et al., 2014, 2016). Ultrasonic detectors mounted on research and fishing vessels that traveled within 166 km of the Mid‐Atlantic coast recorded bats an average of 8.7 km (*n* = 166 passes; range = 1.2–21.9 km; Sjollema et al., 2014), 29.6 km (*n* = 584 passes; range 22.2–44.4 km; Craven et al., 2020), and 60.3 km (*n* = 35 passes; range = 1.2–129.6 km; Peterson et al., 2016; Table 1) from land. A thermal imaging camera (paired with a vertically pointed radar and mounted to a barge) monitored for birds at temporary locations within 0–20 km of the New Jersey coast and detected 45 radar signatures characterized as foraging bats (Geo‐Marine, 2010; Table 1). Nearly two‐thirds (62.5%) of records occurred over water shallow enough to be effectively developed with current technology (i.e., <60 m deep; Tables S1 and S2; Figure 1). Six records occurred over water currently leased for wind development, and six more were in the vicinity of the newly constructed Coastal Virginia Offshore Wind Project (Figure 1). In summary, bats have been seen and detected over a wide area of the Mid‐Atlantic and Gulf of Maine, occurring in areas currently developed for wind and projected for future offshore wind energy development (Musail et al., 2016).

**TABLE 1:** Summary of remote sensing and bat acoustic surveys conducted by ship in the Mid‐Atlantic

Source	Coverage	Distance from land (km)	Number of detectors	Height (m)	Survey dates	Number of nights	Total passes	Overall rate^a^
Geo‐Marine (2010)	Barge moved among grid cells off New Jersey	0–20	n/a^b^		03/24/08‐05/11/08, 10/1/08‐10/19/08, 05/11/09‐05/12/09	56	45	0.80
Sjollema et al. (2014)	Five research and fishing vessels sailed routes off New Jersey and Delmarva Peninsula	1.2–166.0	10 (2 per vessel)	1.8–12.2	03/11/09‐10/31/09, 04/21/10‐08/26/10	86	166	1.93
Peterson et al. (2016)	NOAA research vessel sailed routes from coastal Massachusetts to North Carolina	5.26–129.6	1	10	08/15/14‐09/29/14	52	35	0.67
Craven et al. (2020)	Research vessel sailed routes within BOEM Renewable Energy Lease Area OCS‐A 0512 off New Jersey	22.2–44.4	1	23	05/29/18‐12/02/18	188	584	3.10

Abbreviation: BOEM, Bureau of Ocean Energy Management.

^a^ Passes/detector‐night.

^b^ Acoustic detectors not used. A thermal camera paired with a vertically pointed radar recorded signatures of foraging bats.

To our knowledge, North American bats have never been seen or acoustically detected flying over the Pacific Ocean. Hoary bats are believed to migrate south along the Pacific Coast in autumn (Brown, 1935; Dalquest, 1943), and several species are known to occupy Pacific islands (San Juan Islands, Dalquest, 1940; Vancouver Island, Dalquest, 1943; Haida Gwaii, Burles et al., 2014; Channel Islands, Brown & Rainey, 2018). Hoary bats and western red bats (*Lasiurus blossevillii*) have been documented on Southeast Farallon Island, located approximately 32 km off the northern California coast, using it as a migratory stopover for the past four decades (Cryan & Brown, 2007; Tenaza, 1966). Genetic evidence recently identified a juvenile eastern red bat on Santa Cruz Island, 32 km off the southern California coast (P. Brown & W. Rainey, unpublished data). Combined with genetic confirmation of four specimens in southern California museums (D. Fraser, unpublished data), this extends the current known distribution of eastern red bats by approximately 800–1200 km (Geluso & Valdez, 2019; Solick et al., 2020). Hoary bats are the only extant bat species to colonize the Hawaiian Islands, where reproductive isolation and morphological differentiation after multiple dispersal events led to the formation of a new species, the Hawaiian hoary bat, *Lasiurus semotus* (ʻōpeʻapeʻa; Pinzari et al., 2020; Russell et al., 2015). The presence of bats on offshore islands in the Pacific indicates some movement by bats over the Pacific Ocean, including long‐distance migrating species frequently found as fatalities at land‐based wind facilities. It is unknown whether the lack of observations reflects less activity by bats over the Pacific Ocean or the absence of survey effort by biologists.

### 3.2 Activity rates

Most sightings occurred during daylight hours, and all 38 sightings occurred over a span of 130 years (Table S1), which could imply that offshore bat occurrence is relatively rare. However, multiyear acoustic surveys in the Mid‐Atlantic and Gulf of Maine indicate the nocturnal density of offshore bats and the frequency at which the animals pass by fixed locations at sea is more common, averaging 2.57 passes/night at offshore structures (*n* = 32 site‐years; Table S2; Figure 1; Peterson et al., 2014, 2016). Activity rates of bats recorded at wind turbines and research platforms in the North Sea of Europe averaged 1.01 passes/night (*n* = 7 sites; Table 2). Both sets of activity rates are relatively low and are comparable to rates typically recorded in open, arid regions of the United States (Solick et al., 2020; Weller & Baldwin, 2012), suggesting that bat migration over the ocean is generally dispersed over a relatively wide, featureless area.

**TABLE 2:** Summary of bat acoustic surveys conducted in the North Sea of Europe

Source	Site	Distance from land (km)	Number of detectors	Height (m)	Structure	Survey dates	Number of nights	Total passes	Maximum passes	Overall rate^a^
Lagerveld et al. (2014)	Offshore Wind Farm Egmond aan Zee	15	1	15	Wind turbine	08/29/12‐10/20/12	53	189	70	3.57
	Princess Amalia Windpark	25	1	15	Wind turbine	09/04/12‐09/23/12	20	25	10	1.25
Lagerveld et al. (2015)	Luchterduinen	23	1	15	Wind turbine	03/02/15‐10/09/15	220	11	8	0.05
	Princess Amalia Windpark	25	1	15	Wind turbine	03/23/15‐10/20/15	205	15	7	0.07
Hüppop and Hill (2016)	FINO1	45	1	20	Research platform	08/12/04‐12/31/15	3530	317		0.09
Brabant et al. (2019)	Thorntonbank Wind Farm	27	7	16	Wind turbine base	08/08/17‐11/30/17	798	142		0.18^b^
			4	93	Wind turbine nacelle	08/08/17‐11/30/17	456	9		0.02^b^

^a^ Passes/detector‐night.

^b^ Detectors programmed to only record bats that emitted echolocation calls >30 kHz.

The standard deviation (3.51 passes/night) and the range (0–14.49 passes/night; Table S2) for North American bat acoustic activity data are quite broad, reflecting relatively high interannual variation in activity rates within and between sites. For example, during five years of acoustic monitoring at Matinicus Rock, located 32.9 km off the Maine coast, annual activity rates ranged between 0.41 and 12.06 passes/night, and the maximum number of passes recorded within a single night each year ranged between 21 and 326 passes/night (Table S2). Acoustic data provide an index of bat activity, not abundance (Barclay, 1999), so a high number of passes may represent foraging or exploratory behavior by a few bats and not a large number of individual bats flying by the detector. Regardless, the relatively high degree of variation indicates that bat activity in proximity to structures in the offshore environment is uneven between years, suggesting that weather patterns (Cryan & Brown, 2007) or some other stochastic factor likely determine when and how bats encounter offshore structures during migration.

### 3.3 Temporal variation

All of the Atlantic bat sightings occurred during the autumn: eight in August (including the late July‐early August *Myotis* record), 25 in September (11 of which were videographed during aerial surveys on a single morning; Hatch et al., 2013), and four in October (Table S1). On Bermuda, located 1000 km offshore and where long‐distance migrating bats have been blown off‐course by weather, “a minimum of 100 bats is likely to occur during the fall migration (in a normal year) and perhaps half that number during the spring migration” (Van Gelder & Wingate, 1961: 6). Likewise, on Southeast Farallon Island in the Pacific, hoary bats were seen during the autumn months for 36 of 38 years that records were kept (mean ± standard deviation [*SD*] = 8.2 ± 6.6 bats; median = 6) compared to just two years that bats were seen during the spring (Cryan & Brown, 2007; Tenaza, 1966). Acoustic records in the Gulf of Maine (including offshore structures, coastal areas, and islands) support this seasonal timing of offshore bat activity, with >99% of 75,058 recordings made between 15 July and 15 October, despite just 56% of sampling occurring during this period (Peterson et al., 2014). Bat activity peaked in August at offshore structures in the Gulf of Maine and Mid‐Atlantic (mean ± *SD* = 8.0 ± 1.2; range = May–October; Table S2). The timing of bats in the offshore environment coincides with autumn migration and with the period of highest bat fatalities at land‐based wind facilities in North America (AWWI, 2020; Arnett et al., 2008). The general lack of oceanic records during other seasons suggests bats primarily occupy the offshore environment during autumn migration and that risk of exposure at offshore wind facilities is lower during the rest of the year.

Activity rates for bats recorded during acoustic surveys at offshore structures in the Gulf of Maine and Mid‐Atlantic peaked a few hours after sunset (mean ± *SD* = 2.5 ± 1.7 h after sunset), with a range in peak activity between one and 7 h after sunset (*n* = 28 site‐years; Table S2). However, detectors at three offshore structures recorded bats during daylight hours (*n* = 130 passes; Peterson et al., 2016), and sightings of bats in the Atlantic mainly occurred during the day (91.7% of 24 records that recorded time), with several bats seen as late as 11:00–12:00 in the morning and one bat seen an hour before dusk (Table S1). Therefore, while most bats fly over the ocean at night, some bats will be active during daylight hours, likely in search of a place to land.

### 3.4 Species composition

**FIGURE 2:**
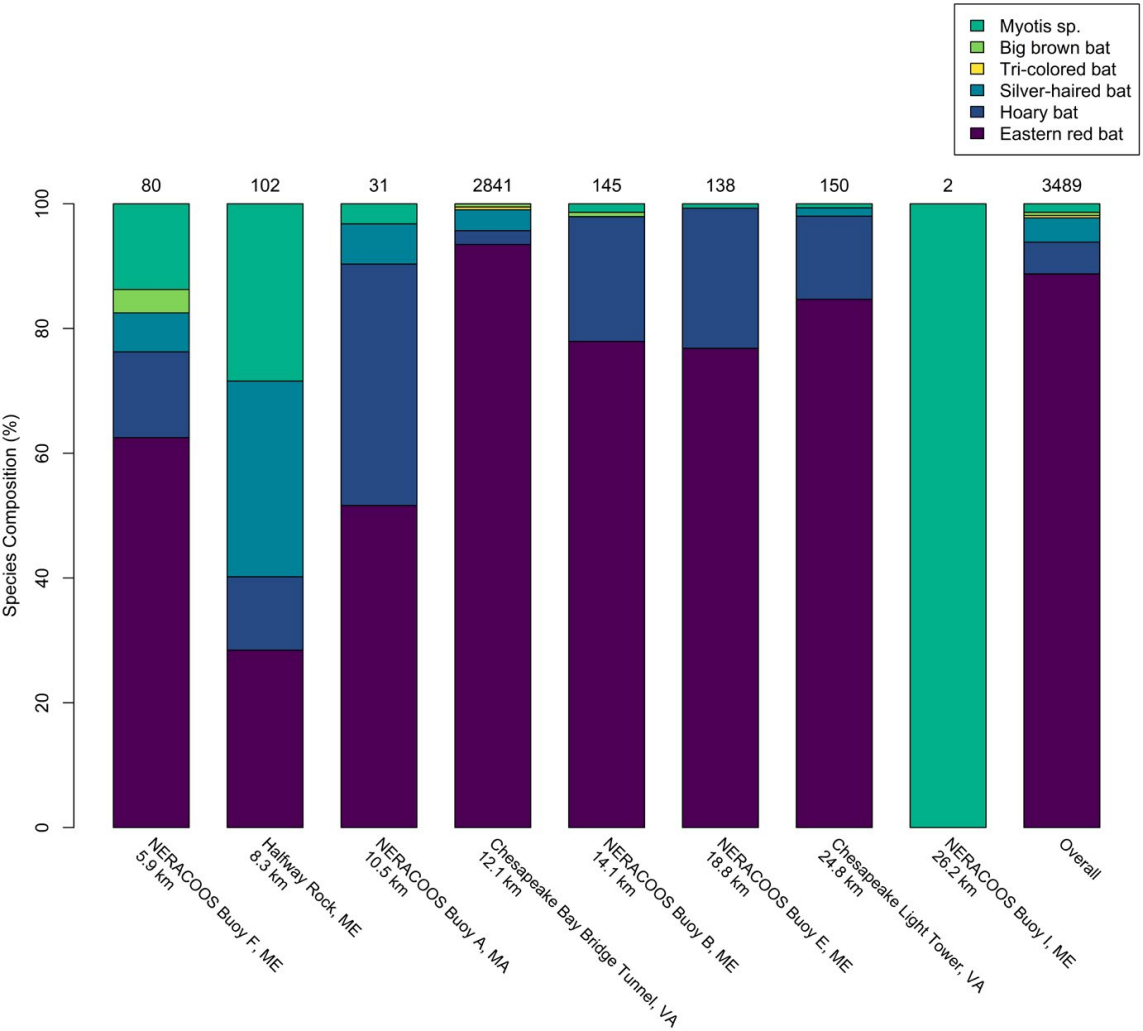
Species composition at offshore structures surveyed with acoustic detectors between 2009 and 2014 in the Gulf of Maine and Mid‐Atlantic, adapted from Peterson et al. (2016) and arranged by distance from land

At least six species of bat have been documented off the Gulf of Maine and Mid‐Atlantic coast (Tables S1 and S2; Figure 2). The vast majority of visual and acoustic records identified to species were of eastern red bats. Silver‐haired bats and hoary bats made up most of the remaining observations, while tricolored bats, big brown bats, and *Myotis* bats were relatively rare. The species composition likely reflects both differences among species in relative abundance and factors that make some species easier or harder to detect.

A review of museum specimens indicates eastern red bats occur throughout coastal areas along the Atlantic Ocean and Gulf of Mexico, extend inland in the spring, and then migrate south along the Atlantic coast in the fall (Cryan, 2003). Telemetry of an eastern red bat along the coast indicates individuals of this species can travel at least 453 km over water in a single night (Dowling et al., 2017). Eastern red bats were reported during 68% of 38 sightings (Table S1) and were the most frequently recorded species (89% of 3489 passes classified to species) at 88% of offshore structures during acoustic surveys in the Mid‐Atlantic and Gulf of Maine (Peterson et al., 2016; Figure 2). Eastern red bats were the main species recorded at 75% of sites (Figure 2), including at NERACOOS Buoy E, located 18.8 km off the shore of Maine. Bats were recorded at this buoy for over 70% of nights in August of 2012, when approximately eight passes/night were recorded on average for nine consecutive nights (Peterson et al., 2016). These data suggest either a pulse of migration past this buoy—possibly evidence of flocking behavior—or that bats were using this buoy as a temporary roost.

Silver‐haired bats were the next most commonly seen species offshore, accounting for 15% of sightings, including one instance when red and silver‐haired bats were observed flying together as part of a large mixed flock (Thomas, 1921; Table S1). As this manuscript was going to publication, we learned of a silver‐haired bat that landed on a charter fishing boat approximately 22 nautical miles southeast of Nantucket Island, Massachusetts, on 25 August 2021 at 0930 (D. O’Dell, personal communication). Acoustic detections of silver‐haired bats were less frequent than for red bats (5%; Figure 2). Some silver‐haired bats apparently do not migrate, with individuals found hibernating in Minnesota and Michigan (Beer, 1956; Kurta et al., 2017), and others overwintering in moderate Pacific Northwest climates (Izor, 1979; Nagorsen et al., 1993). Specimens for silver‐haired bats have been collected during the autumn on the Atlantic coast, indicating coastal migration in the east (Cryan, 2003). Silver‐haired bats were detected at 63% of offshore structures, though were more frequently recorded at sites located closer to land (Figure 2), suggesting that this species migrates relatively close to shore, at least at relatively low altitudes.

Hoary bats have not been seen flying over the ocean (Table S2). Robust data are lacking, but museum records and stable isotope analysis suggest hoary bats migrate from the interior of the country to the coasts in search of more moderate climates and potentially do not actually engage in pronounced latitudinal migration (Cryan, 2003; Cryan et al., 2014). Indeed, there is some evidence that at least some hoary bats can potentially hibernate for all or part of the winter in habitats with stable, nonfreezing climates (Marín et al., 2020; Weller et al., 2016). Three male hoary bats captured in northern California and tracked over the fall and winter using miniature GPS tags and data loggers exhibited a variety of movement patterns, but none of the bats were recorded flying over the Pacific Ocean (Weller et al., 2016).

Despite the general lack of visual records, acoustic surveys indicate hoary bats were widespread in the Atlantic Ocean, present at 88% of offshore structures, though hoary bats were infrequently recorded (4% of passes; Figure 2). Hoary bats are strong, long‐distance fliers, and produce a distinct echolocation call, so it is surprising that members of this species are not more frequently observed or acoustically detected over the Atlantic Ocean. Hoary bats have routinely been observed during the autumn on Bermuda (Allen, 1923; Van Gelder & Wingate, 1961) and have been collected as far away as Iceland (Hayman, 1959), the Orkney Islands (Barrett‐Hamilton, 1910), Southampton Island (Hitchcock, 1943), and Newfoundland (Maunder, 1988), so it is likely that some hoary bats do migrate over the Atlantic, though perhaps not to the same extent as eastern red and silver‐haired bats. Hoary bats have been documented flying at altitudes of 2,400 m above sea level during autumn (Peurach, 2003) and can forego echolocation or produce undetectable echolocation “micro calls” in flight (Corcoran et al., 2021; Corcoran & Weller, 2018), so it is possible that hoary bats (and other species) are more common offshore but potentially fly too high or too quietly to be seen or detected.

As with land‐based wind development, it appears that long‐distance migrating bats are the species most at risk from offshore development. However, species that do not migrate long‐distances, such as tricolored bat (*Perimyotis subflavus*; but see Fraser et al., 2012) and big brown bat (*Eptesicus fuscus*), have been detected acoustically up to 12–14 km from shore at locations in the Gulf of Maine (Figure 2; Peterson et al., 2016). *Myotis* bats were the most widespread species detected acoustically in the Gulf of Maine, being detected on all eight offshore structures for which species data were provided, and *Myotis* were the only species detected (*n* = 2 passes) at the most distant structure, NERACOOS Buoy I, located 26.2 km from land (Figure 2). Echolocation calls by *Myotis* can be confused with steep calls made by eastern red bats (Britzke et al., 2013), so it is possible some of these calls were misclassified. *Myotis* species were more active at structures closer to shore, with 83.3% recorded at structures 8.3 km or less from shore (Figure 2). Sjollema et al. (2014) recorded *Myotis* species up to 11.5 km from shore on research and fishing vessels in the Mid‐Atlantic. Telemetry of a little brown bat (*Myotis lucifugus*) captured on the island of Martha's Vineyard, Massachusetts, found that the bat traveled at least 78 km to a mainland location on Cape Cod, which required some overwater travel (Dowling et al., 2017). One ship record indicates that *Myotis* species are capable of traveling much further from shore. Thompson et al. (2015) describe dozens of unknown *Myotis* bats (probably *M*. *lucifugus*) landing and roosting on their ship as well as on tall “high flier” buoys in the region, 110 km from the nearest land (Table S1; Figure 1). This event occurred in late July or early August, and the bats were believed to have been feeding on relatively large numbers of biting flies present at the time. In the Baltic Sea, approximately 36% of observations at sea (*n* = 1062) were of nonmigratory species feeding on flying insects and apparently gleaning amphipods from the water surface (Ahlén et al., 2009).

### 3.5 Group size

Bats were seen flying alone for 79% of records (Table S1), suggesting that offshore migration is largely a solitary activity. Several records reported large groups of bats flying together in the “dozens,” or estimated at 100–200 individuals (Table S1). All of the records for large groups of long‐distance migrating species were from 1949 or earlier. Mearns (1898) reported “great flights” of eastern red bats over land during autumn in the Hudson Valley of New York. Loose aggregations of eastern red bats during autumn have also been reported migrating over land in Washington, D.C. (Howell, 1908), while concentrations of this species in southern states were noted by Baker and Ward (1967), LaVal and LaVal (1979), and Saugey et al. (1989). It is unknown whether this flocking behavior no longer occurs due to apparent population declines (Winhold et al., 2008), or whether eastern red bats continue to gather and flock in the autumn, unobserved at night. The 11 eastern red bats reported over a three‐hour period on a single morning by Hatch et al. (2013; Table S1), though flying singly, seem reminiscent of Howell's observation from a century earlier. However, all of these reports of apparent group size for bats were made during the daytime, which may not be representative of typical nighttime migration behavior.

### 3.6 Sex and age

Only 11 oceanic records noted the sex of captured or collected individuals: Six bats were male and five were female (Table S1). Age was not specified. Presumably, bats susceptible to collision with offshore installations would comprise adult and juvenile bats of both sexes, as they do on land.

### 3.7 Flight height

None of the records from ships state the precise height at which bats were seen flying, though Murphy and Nichols (1913: 7) describe bats flying “about a gun‐shot” above the sea, Griffin (1940) notes a bat “flew within 15 or 20 feet” (4.5–6.0 m) and A. Rabon and J.B. Thornton (personal communication) photographed an unknown *Lasiurus* circling their boat at approximately 9 m (Table S1). Bats migrating over the Baltic Sea were most often seen flying <10 m above the water surface (Ahlén et al., 2009), including the common noctule bat (*Nyctalus noctula*), which is normally a high‐flying species over land (Ahlén et al., 2007). Nathusius’ pipistrelles (*Pipistrellus nathusii*) were seen flying at heights between 3 and 20 m during ship‐based surveys on the North Sea (Boshamer & Bekker, 2008; Lagerveld et al., 2014). Bats flying low over water have reduced flight costs (“aerodynamic ground effect”; Johansson et al., 2018) and can potentially also use echolocation to remain oriented with the water surface (Ahlén et al., 2009). North American bats flying over the ocean in a similar manner would be less likely to encounter turbine blades. However, bats have been observed ascending rapidly when encountering vertical structures, such as ships, lighthouses, or wind turbines (Ahlén et al., 2009). Off the Atlantic coast of the United States, eastern red bats have been estimated flying 100 – 200 m and >200 m over the ocean based on parallax measurement of aerial video (Hatch et al., 2013; Table S1). Five of the six bats estimated at these heights were videographed in the vicinity of the recently built Coastal Virginia Offshore Wind Project, whose turbine blades reach 222 m above sea level (Table S1; Figure 1). Long‐distance migrating bats in general are capable of flying at altitudes up to at least 460 m (silver‐haired bat) to 2400 m (hoary bat) above sea level as evidenced by collisions with aircraft (Biondi et al., 2013; Peurach, 2003; Peurach et al., 2009). Bats have been recorded flying at nacelle height (93 m) at an offshore wind farm in the North Sea, albeit at a much lower rate (0.02 bats/night) than bats recorded at the base of turbines (16 m; 0.18 bats/night; Brabant et al., 2019; Table 2). These detectors could only record bats emitting echolocation pulses >30kHz (Brabant et al., 2019), which likely reduced the overall bat activity recorded.

### 3.8 Weather

Only 22 (60% of 37) oceanic accounts describe the weather conditions when bats were sighted (Table S1). Three records describe light winds out of the northwest or west‐northwest, while a fourth record mentions an east wind. Four of the accounts took place during periods of relatively calm weather, and the authors suggest that the bats were likely not driven offshore by severe weather. In contrast, the large flock of approximately 200 eastern red bats reported by Carter (1950: 350) was seen on a day with “rain and west‐northwest winds of 20 miles/h” (32.2 km/h). As well, the eastern red bat reported 804.7 km from Nova Scotia by Brown (1953: 139) was “believed by the ship's crew (to) have been driven out to sea by strong winds,” although the actual weather conditions were not described. Sjollema et al. (2014) and Craven et al. (2020) found that bat activity off the mid‐Atlantic coast decreased with increasing wind speeds, a relationship that has also been found in the Baltic Sea (Ahlén et al., 2007), on Assateague Island (Johnson et al., 2011), and at multiple land‐based wind energy studies (Arnett et al., 2008; Baerwald & Barclay, 2011; Horn et al., 2008; Reynolds, 2006; Weller & Baldwin, 2012). That said, Hatch et al. (2013) reported bats flying with tailwinds between 8.9 and 10.1 m/s (*n* = 12 records; Table S1), indicating that bats are capable of flying at relatively high wind speeds offshore.

### 3.9 Offshore wind development

It is unknown what impact, if any, that offshore wind development might have on bat populations or whether any mitigation is needed. In the absence of empirical data, the similar species composition and patterns of bat activity in onshore and offshore environments suggest that bats flying offshore are at some risk of collision. To date, no fatalities of bats have been documented at offshore wind energy facilities worldwide. However, searching for carcasses beneath offshore turbines is not possible, and monitoring of offshore turbines using camera technologies (e.g., thermal, near infrared) that could witness collisions is at very early stages of development and has only been recently pilot‐tested (Brown‐Saracino, 2018; Good & Schmitt, 2020; Matzner et al., 2020; Normandeau Associates, 2021). It is unknown what the potential population impacts could be to bats from offshore wind development. The population size for long‐distance migrating bat species is poorly understood, and it is unclear what proportion of bats move over water as opposed to land. Taken alone, the relatively low numbers of oceanic records in the literature (Table S1) could imply offshore migration is generally a rare event. Yet the acoustic recordings described in this review (Table 1, Table S2) indicate regular, albeit unconcentrated, movement of bats over open water, at least in the Gulf of Maine and the Mid‐Atlantic. What is known is that the vast majority of offshore bat records are of long‐distance migrating bats and occur during autumn migration, the period when the highest fatality rates of these same species at land‐based wind turbines in North America have been recorded (AWWI, 2020). It is prudent to assume that bats flying offshore are at similar risk of collision with turbine blades as conspecifics flying over land. Then again, if offshore wind speeds are typically greater than wind speeds on land, it is possible that bats flying over the ocean area at less risk of collision.

Offshore turbines could be more attractive to bats than mainland turbines. Solick, Pham, et al. (2020) found that bat activity rates increased in a location after turbines are built, and Cryan and Brown (2007) and Baerwald (2018) hypothesized that bats could be attracted to prominent landmarks such as turbines in an otherwise featureless landscape. Some wavelengths of light are attractive to some European migratory species (Voigt et al., 2017, 2018), and the contrast of bright lights against a dark ocean could potentially amplify this attraction. Exploratory behavior by bats to investigate potential landing spots, evaluate feeding opportunities (e.g., Brabant et al., 2019; Hüppop & Hill, 2016), or inspect novel structures on the landscape could increase the probability of collision with moving turbine blades. Prior to landing on ships, bats were observed circling vessels on three occasions (Table S1), presumably inspecting the vessel before landing or moving on. Thermal video at a wind farm in Indiana captured 993 bat detections, of which 88% exhibited “focal” exploratory behaviors, including close approaches to the tower, nacelle, or blades, and the bats often approached multiple times over a period of several minutes (Cryan, Gorresen, et al., 2014).

Offshore structures can provide shelter from adverse weather or an opportunity to rest after a long flight. Indeed, for 12 of the 19 records by ship (63.2%), observers describe bats landing on the rigging, on other parts of the ship, and even on people (Table S1), presumably from exhaustion. On two occasions, bats remained aboard until the ship returned to harbor (Table S1). In the North Sea, bats have been found roosting on offshore installations (Boshamer & Bekker, 2008; Hüppop & Hill, 2016; Petersen et al., 2014), and the animals are likely using structures as temporary refugia during migration. In both the Baltic and North Seas, bats have been found roosting in the nacelles of turbines (Ahlén et al., 2007, 2009), as well as in a transformer station (Lagerveld et al., 2016), inside turbine foundations (Brabant et al., 2019), and in the maintenance equipment on a turbine service platform (Brabant et al., 2019).

Offshore structures can potentially also provide feeding opportunities for migrating bats. Nathusius’ pipistrelle exhibits a “fly‐and‐forage” strategy during autumn migration along the coast of Latvia (Šuba et al., 2012), and North American long‐distance migrating bats feed during autumn migration as well (Reimer et al., 2010; Valdez & Cryan, 2013), including in the vicinity of wind facilities (Foo et al., 2017; Reimer et al., 2018). Migratory and nonmigratory bats were regularly observed foraging on high densities of insects at wind farms located 9.1–14.2 km off the coast of Sweden. Chironomids of marine origin were common offshore, as were terrestrial insects that had flown or drifted from neighboring countries, including ballooning spiders (Ahlén et al., 2007, 2009). So‐called “bioflows” of “aerial plankton” containing trillions of insects amounting to thousands of metric tons of biomass (Hu et al., 2016; Satterfield et al., 2020) can sometimes occur over the open ocean (Alves et al., 2018) and can potentially provide strong incentive for insectivorous bats to seek out and/or follow. The occurrence of “dozens” of *Myotis* bats—not typically associated with long‐distance flight—110 km offshore for a 24‐h period (Table S1), ostensibly feeding on large numbers of biting flies, may be an example of North American bats exploiting a bioflow.

Bats have been observed foraging in close proximity to turbine blades over land and over water (Ahlén et al., 2009; Horn et al., 2008). “Hill‐topping” is a behavior whereby insects follow a hill (or other tall structure) upward and congregate at the top (Shields, 1967). Applied to turbines, this could place foraging bats within proximity of spinning blades (Rydell et al., 2010). Lidar mounted on the nacelles of land‐based turbines and paired with bat detectors documented nightly insect swarms and bat feeding activity (Jansson et al., 2020). Insects are most abundant during nights with low wind speeds, and bats are also most active on nights with low wind speeds (Baerwald & Barlcay, 2011). Thus, as with land‐based facilities, risks to bats at offshore wind facilities may be greatest to long‐distance migrating bats on low wind speed nights.

Foraging bats may also be attracted by marine organisms in the open ocean. Ahlén et al. (2009) observed two species of bats regularly dipping into the water with their feet and hypothesized the bats were gleaning the numerous and widespread amphipods. In the Gulf of California, the fish‐eating bat (*Myotis vivesi*) feeds on fish and crustaceans captured in the ocean (Otálora‐Ardila et al., 2013). In the San Juan Islands, Washington, Yuma bats (*M. yumanensis*), and California bats (*M. californicus*) were shot and collected while flying low and dipping into saltwater off the coast (Dalquest, 1940). It is possible these bats were also foraging on marine organisms.

Given bat use of offshore structures—including turbines—as temporary roosts, and the potential abundance and availability of insects at wind farms, it is possible that offshore creation of roosting and foraging habitat could benefit bat populations. However, roosting and foraging in the vicinity of turbine blades could increase exposure and risk of collision with turbine blades (Peterson, 2020). Turbines located offshore may pose additional risks to bats compared to mainland counterparts. Bat fatalities increase with turbine height (Barclay et al., 2009), and offshore turbines are taller than land‐based turbines (Musail et al., 2016). As noted earlier, bats fly during daylight hours over the ocean, and if this behavior is more common than on land, they may be at greater risk of colliding with offshore turbines throughout the 24‐h period. Lastly, bats have collided with lighthouses, buildings, and television towers during periods of fog or low ceiling height (Cryan & Brown, 2007; Saunders, 1930; Van Gelder, 1956). These weather factors are more common at sea and can potentially increase the risk of collision for bats with offshore turbines.

At present, it is not possible to estimate fatality rates for bats at offshore facilities, and technologies to monitor activity and assess risk are limited. Radar has been used to monitor bat movements over the Baltic Sea, but could only track the large‐bodied common noctule bat (average mass = 30 g; Ahlén et al., 2007). The thermal imaging camera and vertically pointing radar used off the coast of New Jersey only documented 45 bats during approximately 520 h of surveys due to the limited field of view and inability to reliably distinguish commuting bats from birds (Geo‐Marine, 2010). Impact sensors within rotor blades can reliably detect collisions with 57 g tennis balls fired from an air cannon (Hu et al., 2018), but it is unknown if these sensors can detect collisions of long‐distance migrating bats weighing 8–40 g. Acoustic detectors mounted on offshore structures provide information on species composition, timing, and relative activity rates for bats. More offshore acoustic monitoring needs to take place to better understand offshore bat activity patterns, particularly in the Pacific Ocean. More information is needed on bat activity in the vicinity of operational turbines in North American waters and how frequently bats fly over the ocean during daylight hours. A meta‐analysis of land‐based wind facilities in North America concluded that bat activity rates do not predict fatality rates (Solick, Pham, et al., 2020), so offshore activity rates may not be a good indicator of risk. Lagerveld et al. (2020) evaluated three systems combining radar, thermal cameras, and acoustic detectors to monitor for bats flying near turbine blades, but conclude none of the systems are currently ready for deployment in the offshore environment. However, two Acoustic and Thermographic Offshore Monitoring (ATOM^TM^) systems were recently deployed at the Coastal Virginia Offshore Wind facility as part of a pilot project (Normandeau Associates, 2021).

Acoustic deterrents generating high‐frequency noise audible to bats have been found to reduce overall bat fatalities at land‐based wind facilities in North America by up to 62% (Hein & Straw, 2021; Romano et al., 2019; Schirmacher, 2020; Weaver et al., 2020). However, this reduction of bat fatalities varies widely by technology and region, and between years, and between species. For example, during three years of study at a facility in Illinois, a General Electric deterrent reduced bat fatalities by approximately 30% in 2014 and 2015, but no reduction was observed in 2016 (Romano et al., 2019). In Texas, an NRG Systems deterrent reduced fatality rates of hoary bats and Mexican free‐tailed bats (*Tadarida brasiliensis*) by 78% and 54%, respectively, but had no effect on fatalities for northern yellow bats (*L. intermedius*; Weaver et al., 2020), a species frequently found at wind facilities in the southwestern United States (8.1% of fatalities; AWWI 2020). Eastern red bats appear to be the main species at risk of collision with offshore turbines in the Atlantic, but none of the deterrent systems reliably reduced fatality of this species when it was present at a facility. As such, acoustic deterrents by themselves do not appear to be a currently viable minimization strategy at offshore wind facilities. Other deterrents, such as illuminating turbines with dim ultraviolet light (Gorresen et al., 2015) and texture coating (Bennet & Hale, 2018), are currently being tested (Hein & Straw, 2021).

Adjustments to turbine operations can potentially be the most effective minimization strategy for reducing impacts to bats offshore. Land‐based wind facilities in North America have tested raising the turbine cut‐in speed (i.e., the wind speed at which blades rotate and wind‐generated electricity enters the power grid) from the manufactured speed (usually 3.0–4.0 m/s) by 1.5–3.0 m/s, a process known as curtailment (Arnett et al., 2013). It is estimated that total bat fatalities are reduced by 33% for every 1.0 m/s increase in cut‐in speed, for total reductions of 33–79% in a given year at a cut‐in speed of 5.0 m/s (Whitby et al., 2021). Economic analyses of land‐based facilities in North America suggest this type of operational minimization is likely to result in <2–5% energy production loss (Arnett et al., 2011, 2013; Baerwald et al., 2009; Dowling, 2018; Martin, 2015; Whitby et al., 2021). Modeling of theoretical offshore wind facilities in the Atlantic indicates that standard curtailment for bats would result in ≤1.12% decrease in energy production and ≤0.88% revenue losses based on local marginal price data (Dowling, 2018). Wind speeds are generally greater offshore, so low wind speeds (e.g., <5.0 m/s) associated with curtailment would contribute a lower proportion of annual energy production for offshore wind facilities (Dowling, 2018; Eurek et al., 2017). Detection‐based “smart curtailment,” which deactivates turbine blades only when bats are detected during high‐risk periods (e.g., wind speeds <5.0 m/s during August and September), combined with predictive models of offshore bat activity based on regional weather patterns (Smith & McWilliams, 2016), can potentially reduce energy production and revenue losses even further (Hayes et al., 2019). However, because winds tend to be stronger offshore and bats fly at higher wind speeds over the ocean (Hatch et al., 2013; Sjollema et al., 2014; Table S1), operational cut‐in speeds for offshore turbines potentially also need to be increased (and possibly applied during daytime hours) to effectively reduce impacts to bats.

Offshore wind development in the United States is expected to rapidly increase to meet renewable energy initiatives by the end of this decade. Based on the available data, bats occur in the offshore environment and may be susceptible to collision with offshore turbines. There is growing concern that fatalities at terrestrial wind facilities could be impacting North American migratory bat populations. Until we know the population sizes of different bat species, what proportion of those bats move over the ocean, and how many are killed at turbines, it is impossible to determine population‐level impacts and whether operational minimization is needed. However, offshore wind development in North America is at an early stage and has an opportunity to plan for and research potential fatality reduction measures that would be biologically effective as well as economically feasible if offshore bat risk is determined to be significant. Most likely, those fatality reduction measures will include a combination of operational minimization strategies, such as smart curtailment and acoustic deterrents, to effectively harness offshore wind energy generation while reducing potential impacts to bats.

## CONFLICT OF INTEREST

None declared.

## AUTHOR CONTRIBUTION

**Donald I. Solick:** Conceptualization (lead); Data curation (lead); Investigation (lead); Writing‐original draft (lead); Writing‐review & editing (equal). **Christian M. Newman:** Conceptualization (supporting); Funding acquisition (lead); Supervision (lead); Writing‐review & editing (equal).

### OPEN RESEARCH BADGES

This article has earned an Open Data badge for making publicly available the digitally‐shareable data necessary to reproduce the reported results. The data is available at https://doi.org/10.5281/zenodo.5514327.

## Supporting information

Table S1

Table S2

## Data Availability

Tables and figures: Zenodo https://doi.org/10.5281/zenodo.5514327.
