# Supplementary material for: Oceanic records of North American bats and implications for offshore wind energy development in the United States
Source: Ecol Evol. 2021 Oct 11;11(21):14433–47. doi: 10.1002/ece3.8175 (PMC8571582; doi:10.1002/ece3.8175)
Supplement: Supplementary file 1 — Table S1 [file ECE3-11-14433-s001.docx]

| Table S1. Locations and biological information for historic and contemporary oceanic sightings of North American bats. | | | | | | | | | | | | | | |
| --- | --- | --- | --- | --- | --- | --- | --- | --- | --- | --- | --- | --- | --- | --- |
| Map ID | Date | Time | Species | Method | Coordinates | Georeference accuracy | Distance  from land | Water  depth (m) | Number | Sex | Height | Behavior | Weather | Source^a^ |
| 1 | 9/1/1890 |  | silver-haired bat | Collection | 40.95046, -69.432571 | estimate | 56.0 | 24 | 1 |  |  |  |  | NMNH #23604 |
| 2 | 9/1/1890 |  | eastern red bat | Collection | 40.95046, -69.432571 | estimate | 56.0 | 24 | 1 |  |  |  |  | NMNH #23605 |
| 3 | first week of Sept,  1902 | "night" | not specified | Sighting | 37.552099, -76.178499 | estimate | 11.9 | 11 | "a large migration" |  |  | "attempts to alight", "captured on the deck" |  | Allen 1923 |
| 4 | first week  of Sept, 1902 | "night" | not specified | Sighting | 39.124307, -75.225952 | estimate | 11.0 | 8 | "a smaller visitation" |  |  |  |  | Allen 1923 |
| 5 | 9/6/1907 |  | unknown, "little doubt they were Silver-hairs" | Sighting | 40.474917, -73.90645 | estimate | 9.4 | 16 | "a number" |  | "about a gun-shot above the sea" | "struggling toward the Staten Island shore" | "east wind was blowing", "heavy storm clouds hung low on the horizon", "choppy water", "gray,  blustery morning" | Murphy and Nichols 1913 |
| 6 | 8/20/1913 |  | silver-haired bat | Collection | 42.552633, -70.660838 | estimate | 2.6 | 47 | 1 | male |  | "caught with a crab net" |  | MCZ #14874 |
| 7 | 9/7/1918 |  | silver-haired bat | Collection | 40.687628, -72.864876 | estimate | 4.2 | 22 | 1 | male |  |  |  | AMNH #182655 |
| - | 9/1/1920 |  | eastern red bat | Sighting | unknown "3 days out from Philadelphia on our voyage from Cape Town, South Africa" |  |  |  | 1 |  |  | "clinging to the ledge under...giraffe box" |  | Haagner 1921 |
| 8 | 9/3/1919 | 7:07 | eastern red bat | Sighting | 36.970199, -74.856064 | estimate | 94.9 | 54 | 1 |  |  | "darting about ship in erratic fashion, looking for cover", "settled between two booms on the forecastle" | "clear, with a north- west breeze, rather light" | Nichols 1920 |
| 9 | 9/3/1920 |  | eastern red and silver-haired bats | Collection | 35.424689, -75.136965 | estimate | 31.5 | 29 | "a flock of  about a hundred" |  |  | "settled on ship",  "migrating in a considerable flock" |  | Thomas 1921 |
| 10 | 8/17/1929 |  | eastern red bat | Collection | 42, -66 | actual | 161.8 | 98 | 1 | male |  | "flew aboard...and rested on the main sail" | "no offshore gales  have been reported from the region" | Norton 1930 |
| 11 | 8/18/1929 |  | unknown | Collection | 42.117615, -70.257639 | estimate | 6.4 | 60 | 1 |  |  | "boarded" |  | MacCoy 1930 |
| 12 | 9/7/1937 |  | unknown | Sighting | 45.116667, -42.6 | actual | 817.3 | 4747 | 1 |  | "within 15  or 20 feet" |  |  | Griffin 1940 |
| 13 | 8/25/1938 |  | silver-haired bats | Sighting | 39.15, -70.366667 | actual | 235.0 | 2758 | 3 |  |  |  |  | Griffin 1940 |
| 14 | 9/29/1949 | "day break" | eastern red bats | Collection | 40.166667, -71 | actual | 123.7 | 137 | "estimated at about 200" |  |  | "flying about the ship", "some remained on board searching dark places up to the time the ship reached the  port" | "overcast with some rain and the wind was west-northwest at 20 miles per hour" | Carter 1950 |
| 15 | 10/7/1952 |  | eastern red bat | Collection | 42.7, -62.966667 | actual | 196.9 | 1402 | 1 | female |  |  | "may have been driven out to sea by  strong winds" | Brown 1953 |
| 16 | 8/19/1953 | 11:00 | silver-haired bat | Collection | 39.6, -71.05 | actual | 176.2 | 2317 | 1 | male |  | "circled the ship  several times before coming to rest" | "light and variable 5- 10 knot NW winds" | Mackiewicz and Backus 1956 |
| 17 | 8/25/1953 | 11:00 | eastern red bat | Collection | 39.633333, -70.316667 | actual | 180.7 | 2203 | 1 | male |  | "captured in the rigging after...brief flight  around the vessel" | "WNW winds of 5-10 knots prevailed" | Mackiewicz and Backus 1956 |
| 18 | 9/12/1962 | 12:00 | eastern red bat | Collection | 42.25, -67.516667 | actual | 201.6 | 260 | 1 | female |  |  |  | MCZ #50177 |
| 19 | 9/24/1964 | - | eastern red bat | Collection | 36.2, -75.35 | actual | 33.6 | 31 | 1 | - |  |  |  | AMNH #208650 |
| 20 | mid- October, 1969 | "early morning " | eastern red bat | Collection | 42.5, -66.166667 | actual | 111.9 | 227 | 1 | female |  |  |  | Peterson 1970 |
| 21 | "over 2 days in late July or early August 2003" | "an hour before dusk" | Myotis, probably lucifugus | Sighting | "Gulf of Maine, about 1- 10 km sw of a feature known as Southwest Bank" | estimate | 69.8 | 160 | "dozens" |  |  | "dozens circled about" "landed on author" "dozens hanging in rigging, wheelhouse, and living quarters" "near daylight everywhere on vessel by the dozens" "also roosting in high flier (large buoys) within 2 - 8 km" "by nightfall had  dispersed" | "clear with warm temperatures and no wind" "no storms in area" "maximum wind speed was 12.2 km/hr with only 4 hourly records of >10km/hr winds" | Thompson et al. 2015 |
| 22 | 11/27 -  12/2/2010 |  | eastern red bat | Sighting | 43.89, -60.2 | actual | 163 | 31 | 3 |  |  | "For 2-to-3 consecutive evenings, up to 3 bats were observed at dusk" | "mainly overcast, good visibility (18 km), temperature 5 to 8°C...predominantly northwest  winds...Beaufort 2-6" | Czenze et al. 2011 |
| 23 | 9/6/2012 | 9:33 | eastern red bat | Sighting | 38.768562, -74.48744 | actual | 39.5 |  | 1 |  | "within normal line of sight" | "flying southeast at a distance of approximately 150 m  from the ship" | 8.9 m/s from SW | Hatch et al. 2013 |
|  |  |  |  |  |  |  |  | 27 |  |  |  |  |  |  |
| 24 | 9/11/2012 | 7:56 | eastern red bat | Video | 38.741295, -74.801508 | actual | 23.6 | 16 | 1 |  | >200 m | flying SW | 9.3 m/s from NW | Hatch et al. 2013 |
| 25 | 9/11/2012 | 8:16 | eastern red bat | Video | 38.715514, -74.809183 | actual | 22.7 | 15 | 1 |  |  | flying SW | 9.3 m/s from NW | Hatch et al. 2013 |
| 26 | 9/11/2012 | 8:19 | eastern red bat | Video | 38.693065, -74.666917 | actual | 34.6 | 22 | 1 |  |  | flying SW | 9.3 m/s from NW | Hatch et al. 2013 |
| 27 | 9/11/2012 | 8:20 | eastern red bat | Video | 38.684703, -74.615381 | actual | 37.9 | 25 | 1 |  |  | flying SW | 9.3 m/s from NW | Hatch et al. 2013 |
| 28 | 9/11/2012 | 8:49 | eastern red bat | Video | 36.808896, -75.648807 | actual | 26.5 | 21 | 1 |  |  | flying W | 9.3 m/s from NW | Hatch et al. 2013 |
| 29 | 9/11/2012 | 9:41 | eastern red bat | Video | 37.014651, -75.451316 | actual | 44.6 | 27 | 1 |  | >200 m | flying SW | 10.1 m/s from N | Hatch et al. 2013 |
| 30 | 9/11/2012 | 9:43 | eastern red bat | Video | 37.027304, -75.517269 | actual | 38.7 | 22 | 1 |  | >200 m | flying SW | 10.1 m/s from N | Hatch et al. 2013 |
| 31 | 9/11/2012 | 9:45 | eastern red bat | Video | 37.055766, -75.63717 | actual | 27.6 | 16 | 1 |  | 100-200 m | flying SW | 10.1 m/s from N | Hatch et al. 2013 |
| 32 | 9/11/2012 | 10:13 | eastern red bat | Video | 37.144471, -75.327618 | actual | 50.3 | 30 | 1 |  | >200 m | flying SW | 10.1 m/s from N | Hatch et al. 2013 |
| 33 | 9/11/2012 | 10:19 | eastern red bat | Video | 37.237921, -75.592406 | actual | 25.8 | 17 | 1 |  | >200 m | flying SW | 10.1 m/s from N | Hatch et al. 2013 |
| 34 | 9/11/2012 | 10:39 | eastern red bat | Video | 38.517294, -74.663008 | actual | 33.6 | 19 | 1 |  |  | flying NW | 10.1 m/s from N | Hatch et al. 2013 |
| 35 | 8/25/2017 | 10:30 | eastern red bat | Photograph | 41.698298, -69.749989 | actual | 15.4 | 62 | 1 | female |  | "landed on boat" "stayed on boat all day and still on boat when it returned in the evening" "gone in the  morning" |  | B. Perkins and  D. O'Dell, pers comm. |

| 36 | 10/19/2019 |  | eastern red bat | Photograph | "35 nm from Hatteras, se from the inlet, in the Gulf Stream" | estimate | 63.7 | 729 | 1 | female | "flew onto captain's sweatshirt and landed on his arm" "took to shore under care of a vet and was released  the next day" |  | K. Sutherland, pers comm. |
| --- | --- | --- | --- | --- | --- | --- | --- | --- | --- | --- | --- | --- | --- |
| 37 | 10/10/2020 | 10:30 | *Lasiurus* sp. | Photograph | 36.848611, -74.7 | actual | 109.1 | 87 | 1 | ~9 m | "circled boat twice, then buzzed very closely before flying  towards shore" | "wind blowing from southwest at 10-13 mph" | 1. Rabon and J. 2. Thornton, pers comm. |
| ^a^ NMNH = Smithsonian National Museum of Natural History; MCZ = Museum of Comparative Zoology; AMNH = American Museum of Natural History | | | | | | | | | | |  |  |  |

References:

1. Czenze, Z., Wong, S. N. P., and Willis, C. K. R. (2011). Observations of eastern red bats (<i>Lasiurus borealis</i>) 160 km from the coast of Nova Scotia. Bat Research News, 52, 28-30.
2. MacCoy, C. V. (1930). Mammals. Bulletin of the Boston Society of Natural History, 56, 35.
