# Supplementary material for: Oceanic records of North American bats and implications for offshore wind energy development in the United States
Source: Ecol Evol. 2021 Oct 11;11(21):14433–47. doi: 10.1002/ece3.8175 (PMC8571582; doi:10.1002/ece3.8175)
Supplement: Supplementary file 2 — Table S2 [file ECE3-11-14433-s002.docx]

| Table S2. Summary of acoustic recordings at offshore structures in the Mid-Atlantic and Gulf of Maine (Peterson et al. 2014; Peterson et al. 2016). | | | | | | | | | | | | | | | |
| --- | --- | --- | --- | --- | --- | --- | --- | --- | --- | --- | --- | --- | --- | --- | --- |
| Map ID | Site | Structure | Coordinates | Height (m) | Distance  from land (km) | Water depth (m) | Year | Survey dates | Nights | Total passes | Maximum passes | Overall rate^a^ | Peak hour after sunset | Peak month | Species diversity |
| *Mid-Atlantic* | | | | | | | | | | | | | | | |
| A | Albermarie ATON, NC | Beacon | 36.04506, -76.0014 | 3 | 7.9 | 6 | 2012 | 06/20-12/31 | 195 | 647 | 67 | 3.32 | 3 | 9 | 4 |
|  |  |  |  |  |  |  | 2013 | 01/01-08/02 | 183 | 51 | 24 | 0.28 | 4 | 7 | 3 |
|  |  |  |  |  |  |  | 2014 | 02/28-12/31 | 137 | 279 | 33 | 2.04 | 3 | 10 | 6 |
| B | Chesapeake Light Tower,  VA | Tower | 36.90972, -75.7097 | 36 | 24.8 | 12 | 2012 | 04/29-10/25 | 180 | 165 | 144 | 0.92 | 6 | 5 | 1 |
|  |  |  |  |  |  |  | 2013 | 01/01-12/31 | 365 | 71 | 13 | 0.19 | 6 | 9 | 3 |
|  |  |  |  |  |  |  | 2014 | 01/01-11/30 | 326 | 103 | 42 | 0.32 | 1 | 8 | 4 |
| C | Chesapeake Bay Bridge  Tunnel, VA | Building | 37.036669, -76.076649 | 15 | 12.1 | 12 | 2012 | 04/26-11/26 | 215 | 3115 | 234 | 14.49 | 3 | 7 | 4 |
|  |  |  |  |  |  |  | 2013 | 05/17-12/18 | 216 | 1777 | 133 | 8.23 | 3 | 9 | 3 |
|  |  |  |  |  |  |  | 2014 | 03/01-12/15 | 290 | 1474 | 84 | 5.08 | 3 | 8 | 3 |
| *Gulf of Maine* | | | | | | | | | | | | | | | |
| D | NERACOOS Buoy I, ME | Buoy | 44.1, -68.1 | 2 | 26.2 | 101 | 2013 | 06/24-12/31 | 191 | 9 | 3 | 0.05 | 1 | 8 | 1 |
|  |  |  |  |  |  |  | 2014 | 01/01-03/03 | 62 | 0 | 0 | 0.00 |  |  |  |
| E | NERACOOS Buoy F, ME | Buoy | 44.05, -68.99 | 2 | 5.9 | 55 | 2013 | 06/17-09/24 | 100 | 233 | 22 | 2.33 | 3 | 8 | 5 |
| F | NERACOOS Buoy E,  ME | Buoy | 43.71, -69.35 | 2 | 18.8 | 93 | 2012 | 04/11-12/31 | 265 | 378 | 44 | 1.43 | 2 | 8 | 3 |
| G | NERACOOS Buoy B,  ME | Buoy | 43.17, -70.42 | 2 | 14.1 | 63 | 2012 | 04/11-12/31 | 265 | 411 | 32 | 1.55 | 2 | 8 | 3 |
|  |  |  |  |  |  |  | 2013 | 01/01-12/17 | 203 | 93 | 11 | 0.46 | 2 | 8 | 3 |
| H | NERACOOS Buoy A,  MA | Buoy | 42.52, -70.56 | 2 | 10.5 | 64 | 2011 | 06/01-10/15 | 137 | 84 | 9 | 0.61 | 2 | 8 | 3 |
|  |  |  |  |  |  |  | 2012 | 04/14-07/02 | 80 | 7 | 3 | 0.09 | 1 | 5 | 2 |
|  |  |  |  |  |  |  | 2013 | 06/18-08/18 | 62 | 41 | 20 | 0.66 | 2 | 7 | 3 |
|  |  |  |  |  |  |  | 2014 | 04/01-04/17 | 17 | 0 | 0 | 0.00 |  |  |  |
| I | Matinicus Rock, ME | Lighthouse | 43.783831, -68.855002 | 14 | 32.9 | 34 | 2009 | 09/02-09/14 | 13 | 102 | 43 | 7.85 | 1 |  | 4 |
|  |  |  |  |  |  |  | 2010 | 08/05-10/31 | 88 | 178 | 24 | 2.02 | 8 | 8 | 3 |
|  |  |  |  |  |  |  | 2012 | 06/22-10/23 | 124 | 1495 | 326 | 12.06 | 2 | 10 | 4 |
|  |  |  |  |  |  |  | 2013 | 06/25-11/04 | 133 | 441 | 161 | 3.32 | 2 | 9 | 4 |
|  |  |  |  |  |  |  | 2014 | 04/04-12/31 | 272 | 111 | 21 | 0.41 | 1 | 8 | 4 |
| J | Mount Desert Rock, ME | Lighthouse | 43.964338, -68.1411 | 17 | 41.6 | 68 | 2009 | 08/17-12/31 | 137 | 597 | 168 | 4.36 | 1 | 8 | 4 |
|  |  |  |  |  |  |  | 2010 | 08/26-12/31 | 128 | 277 | 92 | 2.16 | 2 | 9 | 3 |
|  |  |  |  |  |  |  | 2011 | 01/01-09/17 | 260 | 366 | 133 | 1.41 | 1 | 8 | 4 |
|  |  |  |  |  |  |  | 2013 | 07/09-07/23 | 15 | 7 | 7 | 0.47 | 1 |  | 1 |
|  |  |  |  |  |  |  | 2014 | 08/08-10/27 | 81 | 336 | 77 | 4.15 | 1 | 8 | 3 |
| K | Halfway Rock, ME | Lighthouse | 43.655994, -70.0369 | 23 | 8.3 | 20 | 2009 | 08/13-12/31 | 141 | 287 | 60 | 2.04 | 3 | 8 | 4 |
|  | |  |  |  |  |  | 2010 | 01/01-02/24 | 55 | 0 | 0 | 0.00 |  |  |  |
|  | |  |  |  |  |  | 2013 | 05/31-07/01 | 32 | 3 | 1 | 0.09 |  |  | 1 |
| ^a^ passes/detector-night | |  |  |  |  |  |  |  |  |  |  |  |  |  |  |
